# Effects of rumen-protected lysine on antler growth performance, fecal bacterial community, and blood gene expression in sika deer

**DOI:** 10.3389/fvets.2025.1583605

**Published:** 2025-07-11

**Authors:** Bo Yang, Yuhang Zhu, Yongxiang Li, Yating Gao, Huazhe Si, Zhipeng Li

**Affiliations:** ^1^College of Animal Science and Technology, Jilin Agricultural University, Changchun, China; ^2^Joint International Research Laboratory of Modern Agricultural Technology, Ministry of Education, Jilin Agricultural University, Changchun, China; ^3^Jilin Provincial Engineering Research Center for Efficient Breeding and Product Development of Sika Deer, Jilin Agricultural University, Changchun, China; ^4^Key Laboratory of Animal Production, Product Quality and Security, Ministry of Education, Jilin Agricultural University, Changchun, China

**Keywords:** sika deer, lysine, antler, fecal bacteria, gene expression

## Abstract

**Introduction:**

Velvet antler is an important product of sika deer (*Cervus nippon*), and its growth is closely related to dietary amino acid supplementation. Lysine is one of the major limiting amino acids in animals; however, the mechanism underlying its effect on velvet antler growth in sika deer remains unclear.

**Methods:**

This study investigated the impact of rumen-protected lysine supplementation on velvet antler growth, nutrient digestibility, gut bacteria, serum biochemical parameters, and gene expression in sika deer. Fifteen healthy 2-year-old male sika deer were randomly assigned to three dietary groups: control (0 g/day rumen-protected lysine, CON), low (5 g/day rumen-protected-lysine, LLys), and high (10g/day rumen-protected lysine, HLys).

**Results:**

Supplementation with rumen-protected lysine significantly increased antler weight and dry matter, crude protein, neutral detergent fiber, and acid detergent fiber digestibility (*P* < 0.05). The concentrations of acetate and propionate in the feces of the LLys were significantly higher than those in the CON (*P* < 0.05). The relative abundance of *Fibrobacter* in the feces was significantly higher in the HLys group compared to the CON group (*P* < 0.05). The relative abundance of *Papillibacter, Coprococcus, Anaerorhabdus furcosa*, and *Parabacteroides* were significantly lower in the HLys than in the CON (*P* < 0.05). The gene expression was influenced in both HLys and LLys groups compared to the CON group, with upregulated dierentially expressed genes (DEGs) and downregulated DEGs identified. KEGG pathway analysis showed upregulated DEGs were enriched in MAPK, PI3K-Akt, TNF, p53, FoxO, JAK-STAT, NF-κB, and Toll like receptor signaling pathways. Down regulated DEGs were enriched in glutathione metabolism, lysine degradation, fatty acid elongation, biosynthesis of unsaturated fatty acids, Th17 and Th2 cell differentiation.

**Conclusion:**

Overall, these results provide novel insights into the effect of rumen-protected lysine supplementation on antler growth, host digestibility and metabolism, fecal microbiota, and blood transcriptome of sikadeer.

## 1 Introduction

Since deer antlers are highly valued in traditional Chinese medicine, sika deer (*Cervus nippon*) are intensively farmed for antler production (1). Although China has over a million sika deer to produce antlers, meeting the demands of the rapidly expanding antler market remains challenging (2). Antlers are rapidly growing bone tissue, with growth rates reaching 1.25 cm/day in sika deer, and primarily composed of proteins and mineral elements such as calcium (3, 4). Therefore, enhancing the supply of proteins and mineral elements is essential for supporting increased antler production. Lysine is an essential limiting amino acid in corn-soybean meal-based feed, and is essential for protein digestion and calcium absorption in the gastrointestinal tract (5). Currently, only one study has investigated the effects of rumen-protected lysine on juvenile fallow deer (*Dama dama*), which showed that rumen-protected lysine did not significantly promote the first antler growth (6). However, the effect of rumen-protected lysine on antler growth performance in adult sika deer is still not well understood. Dietary rumen-protected lysine supplementation has been shown to enhance the amino acid absorption and utilization and thus increase the milk protein content in dairy cows, or promote average daily gain in sheep (7, 8). Previous findings indicate that lysine positively influences the production performance in adult ruminants by enhancing protein digestion and calcium absorption. Based on these functions, we hypothesized that lysine supplementation may also contribute to improved antler growth in sika deer.

Moreover, supplementation with rumen-protected lysine has been reported to influence the fecal bacterial composition and community, increasing the relative abundance of *Christensenellaceae R-7* and *Oscillospiraceae UCG-005* while reducing the abundance of *Turicibacter*, thus suggesting that rumen-protected lysine may affect intestinal fiber degradation and utilization (9). Furthermore, studies have also found that lysine directly or indirectly affects the activation of MAPK (10), PI3K-Akt (11) and NF-κB (12) signaling pathways through histone modification to regulate the gene expression of cell proliferation, metabolism and immune functions (13). Therefore, we hypothesized that lysine supplementation could also affect the composition of intestinal bacteria and the expression of blood genes in sika deer.

In this study, we aimed to investigate: whether rumen-protected lysine supplementation (i) alters velvet antler growth performance, protein and calcium utilization and serum parameters (ii) influences fecal bacterial features and metabolites and (iii) affects gene transcription in the blood of sika deer.

## 2 Materials and methods

### 2.1 Experimental design

The experiment was carried out at Jilin Zhongte Agricultural Technology Co., Ltd. in Jilin Province, China. Fifteen healthy 2-year-old male sika deer (average body weight = 92.35 ± 12.29 kg) were selected in this study. The sika deer were randomly assigned to one of three dietary groups based on supplementation with rumen-protected lysine: the control group (CON: basic diet, *n* = 5), the low lysine group (LLys: basic diet + 5 g/day rumen-protected lysine, *n* = 5), and the high lysine group (HLys: basic diet + 10 g/day rumen-protected lysine, *n* = 5). Rumen-protected lysine (60% lysine content) was provided by King Techina (Hangzhou, China). The selected doses were based on a previous study using 5 g/day of 50%-content rumen-protected lysine in fallow deer (6), and adjusted to 60% content in our trial to assess both comparable (5 g/d) and higher dose (10 g/d) effects. The animals were individually housed in pens with free access to drinking water and were fed twice daily at 6:00 and 16:00. Each animal received 2.5 kg dry matter daily and a concentrate-to-roughage ratio of 7:3 (Supplementary Table S1). The experimental period lasted 8 weeks after surgical removal of the hard antler button, including a 1-week adaptation period and 7 weeks of dietary treatment (14–16).

### 2.2 Sampling and laboratory analyses

At the end of the experiment, velvet antlers were collected and weighed. The antlers from 15 sika deer were weighed on-site using a high-precision electronic balance (Mettler-Toledo; accuracy: 0.01 g, capacity: 0–10 kg). Before weighing, the balance was calibrated with standard calibration weights, and the tray was tared. Each antler was gently placed on the tray, and the weight was recorded once the reading stabilized. Each antler was measured three times, and the average value was used as the final recorded weight. Blood samples from each animal were collected via jugular vein puncture using vacuum blood collection tubes containing silica particles or dipotassium ethylenediaminetetraacetic acid (EDTA-K_2_). The blood was then centrifuged at 3,500 × *g* for 10 min to obtain serum. Serum concentrations of aspartate aminotransferase, alanine aminotransferase, alkaline phosphatase, total protein, triglycerides, total cholesterol, high-density lipoprotein cholesterol, low-density lipoprotein cholesterol, and blood urea nitrogen were determined using commercial assay kits (Biosino Bio-Technology and Science Incorporation, Beijing, China), and serum-free amino acid concentrations were measured using ultra-high-performance liquid chromatography-mass spectrometry (UHPLC-MS) (Agilent 1290 Infinity II series). Additionally, the blood from the EDTA-K_2_ tubes was mixed with TRIzol™ Reagent (Invitrogen, Carlsbad, CA, USA) at a 1:3 ratio and stored at −80°C for subsequent transcriptome sequencing. Fecal samples were collected to extract volatile fatty acids (VFAs) and their concentrations were quantified using gas chromatography (6890GC, Agilent Technologies, Santa Clara, CA, USA) equipped with a flame ionization detector and a DB-FFAP column. Approximately 3 g of each fecal sample from each animal was collected into 5 mL sterile cryogenic storage tubes and stored at −80°C for bacterial analysis.

### 2.3 DNA and RNA extraction and sequencing

Microbial genomic DNA was extracted from fecal samples by QIAamp^®^ Fast DNA Stool Mini Kit (QIAGEN, Valencia, CA, USA) following the manufacturer's instructions. The bacterial 16S rRNA gene in the V3-V4 region was amplified using primers 515F (5′-GTGCCAGCMGCCGCGGTAA-3′) and 806R (5′-GGACTACHVGGGTWTCTAAT-3′), each incorporating the appropriate Illumina adapter sequence and an 8-bp barcode. The amplicons were then purified with the QIAquick PCR Purification Kit (QIAGEN, Valencia, CA, USA) and sequenced on the Illumina NovaSeq 6000 platform using paired-end reads.

For the blood samples, RNA extraction was performed on 15 samples using the Qiagen RNeasy Mini Kit (QIAGEN, Valencia, CA, USA). RNA concentration and quality were assessed with a NanoDrop 2000 (NanoDrop, Wilmington, DE, USA), and samples with an RNA integrity number (RIN) > 7.0 were selected for library construction. Two samples (one from LLys and one from HLys) with low RNA quality (RIN < 7.0) were excluded, resulting in a total of 13 blood samples (CON:5, LLys: 4, HLys: 4) used for transcriptome sequencing. RNA-Seq libraries were prepared using the NEBNext^®^ UltraTM RNA Library Prep Kit (Illumina, San Diego, CA, USA) with 1.5 μg of RNA from each sample. The libraries were quantified using a Qubit 2.0 Fluorometer and subsequently sequenced on an Illumina HiSeq 4000 platform with 150 bp paired-end sequencing.

### 2.4 Bioinformatic analysis

The 16S rRNA sequences were first assembled into contigs using FLASH (17). The sequences were used to cluster amplicon sequence variants (ASVs) with a 100 % similarity cutoff by DADA2 (18) and UCHIME (19) was used to identify and remove chimeric sequences. Representative sequences of each ASV were matched against the SILVA database (v138.1) (20). Alpha-diversity indices were calculated using QIIME 2. The Principal Coordinates Analysis (PCoA) based on the Bray-Curtis dissimilarity matrix, Jaccard distance, weighted UniFrac distance and unweighted UniFrac distance was performed to illustrate differences in fecal bacterial communities among three groups, and the *P*-value was determined using PERMANOVA analysis with 999 permutations. The functional profiles of bacteria were predicted using the phylogenetic investigation of communities by reconstruction of the unobserved states (PICRUSt2), referencing the Greengenes database (21), and gene predictions were summarized according to KEGG pathways.

For the transcriptome analysis, low-quality and adapter sequences were removed using Trimmomatic (22). The clean reads were then aligned to the sika deer reference genome using HISAT2 (23). Differentially expressed genes (DEGs) were identified using the DESeq2 package, with criteria set at |log_2_(FC)| > 0.5 and a false discovery rate (FDR) of < 0.05 after Benjamini and Hochberg correction. Principal Component Analysis (PCA) was conducted to visualize gene expression patterns in blood samples across the groups. KEGG pathway enrichment analysis for differentially expressed genes (DEGs) was performed using KOBAS, applying an FDR threshold of < 0.05 with Benjamini-Hochberg correction (24).

### 2.5 Statistical analysis

This study employed one-way analysis of variance (ANOVA) to assess the statistical significance of differences among the three groups in terms of antler growth performance, serum biochemical parameters, nutrient digestibility, serum amino acids, and volatile fatty acids. Prior to ANOVA, the normality of the data distribution was tested using the Shapiro-Wilk test (α = 0.05), and homogeneity of variance was assessed using Levene's test. For data meeting the assumptions of normality and homogeneity of variance, ANOVA was applied to analyze differences in the aforementioned parameters, with significant results further examined using Duncan's test for multiple comparisons. For data that did not meet the assumptions of normality or homogeneity of variance, the Kruskal-Wallis (K-W) test was used to evaluate the significance of bacterial relative abundance and potential functional profiles. Pearson correlation analysis was conducted to determine the relationship between bacterial relative abundance and volatile fatty acid concentrations in feces. Linear regression analysis was used to estimate the association between the final body weight and antler weight. All statistical analyses were performed using SPSS software (IBM SPSS Statistics 26; IBM SPSS Inc., Chicago, IL, USA), with a significance level set at *P* < 0.05.

## 3 Result

### 3.1 Rumen-protected lysine supplementation improved antler weight and nutrient digestibility

The supplementation of rumen-protected lysine significantly increased the final weight of antlers in HLys compared to the CON (*P* < 0.05). The final body weight and antler weight were positively correlated (Supplementary Figure S1A). The digestibility of dry matter (DM), crude protein (CP), neutral detergent fiber (NDF), and acid detergent fiber (ADF) was significantly increased in the HLys group compared to the CON group. Moreover, calcium digestibility showed an increasing tendency after rumen-protected lysine supplementation, but the difference was not statistically significant (*P* > 0.05, Table 1).

**Table 1:** Antler weight, average daily gain, and apparent nutrient digestibility in three groups.

**Item**	**CON**	**LLys**	**HLys**	**SEM**	***P*-value**
Antler weight (AW, g)	875.54^b^	895.33^a^^b^	987.78^a^	22.297	0.08
Daily gain of antler (g/d)	18.52	18.31	20.47	0.470	0.10
Final body weight (FBW, Kg)	94.20	95.13	86.50	3.501	0.60
ADMI (g/d)	2,453	2,654	2,721	81.9	0.43
**Apparent nutrient digestibility**					
DM	86.95^b^	88.96^a^^b^	90.94^a^	7.128	0.04
CP	86.65^b^	88.13^a^^b^	90.22^a^	7.142	0.11
NDF	77.72^b^	82.90^a^	85.20^a^	1.189	<0.01
ADF	40.67^b^	58.88^a^	62.16^a^	4.502	0.02
Ca	66.65	68.70	73.72	1.423	0.17

Values are the mean of five replicates (*n* = 5), with standard error of the mean (SEM).

CON = control group; LLys = with supplementary 5 g/d rumen-protected lysine; HLys = with supplementary 10 g/d rumen-protected lysine. ADMI, Average Dry Matter Intake; DM, Dry matter; CP, Crude protein; NDF, Neutral detergent fiber; ADF, Acid detergent fiber; Ca, Calcium. All nutrient digestibility values are expressed as a percentage of dry matter (% of DM).

a, b means within a row with different superscript letters differ significantly from each other (*P* < 0.05).

### 3.2 Rumen-protected lysine supplementation had minimal effects on serum metabolite concentrations

To further elucidate the effects of rumen-protected lysine supplementation on the digestion of CP and Ca in sika deer, we next focused on changes in serum metabolite levels. The results showed that blood urea nitrogen (BUN) concentration was significantly higher (*P* < 0.05) and Ca concentration exhibited a non-statistically significant increase (*P* > 0.05) in HLys compared to the CON. The concentrations of total protein, albumin and globulin also showed non-significant changes among the three groups (Table 2).

**Table 2:** Comparison of serum biochemical parameters in sika deer from the three groups.

**Item**	**CON**	**LLys**	**HLys**	**SEM**	***P*-value**
BUN (mmol/L)	3.57^b^	4.05^a^^b^	4.23^a^	0.154	0.08
Ca (mmol/L)	1.89	1.91	1.95	0.052	0.94
TG (mmol/L)	0.20	0.28	0.27	0.021	0.22
TC (mmol/L)	2.00	1.97	1.89	0.063	0.88
HDL (mmol/L)	2.75	2.76	2.65	0.077	0.94
LDL (mmol/L)	0.87	0.87	0.85	0.029	0.97
GLU (mmol/L)	9.20	10.18	10.04	0.312	0.43
TP (g/L)	62.9	61.01	61.97	1.000	0.61
ALB (g/L)	32.06	32.04	31.45	0.561	0.98
ALP (U/L)	284.28	245.45	274.09	25.113	0.80
GLB (g/L)	30.84	28.98	30.52	0.978	0.58
ALT (U/L)	31.29	32.85	37.6	1.627	0.41
AST (U/L)	67.29	62.76	63.78	2.064	0.70

Values are the mean of five replicates (*n* = 5), with standard error of the mean (SEM).

CON = control group; LLys = with supplementary 5 g/d rumen-protected lysine; HLys = with supplementary 10 g/d rumen-protected lysine. BUN, Blood Urea Nitrogen; Ca, Calcium; TG, Triglycerides; TC, Total Cholesterol; HDL, High-Density Lipoprotein Cholesterol; LDL, Low-Density Lipoprotein Cholesterol; GLU, Glucose; TP, Total Protein; ALB, Albumin; ALP, Alkaline Phosphatase; GLB, Globulin; ALT, Alanine Aminotransferase; AST, Aspartate Aminotransferase.

a, b means within a row with different superscript letters differ significantly from each other (*P* < 0.05).

Due to the changes in BUN, we hypothesized that serum-free amino acid levels might have changed in the sika deer. We next measured free amino acid concentrations in the serum of the three groups. The results showed that the addition of rumen-protected lysine did not significantly change the serum-free amino acid concentrations in both LLys and HLys groups (*P* > 0.05, Table 3). Although not statistically significant, most serum free amino acids, including lysine, showed numerical increases in the LLys and HLys groups.

**Table 3:** Comparison of free amino acids in the serum of sika deer from the three groups.

**Item (nmol/L)**	**CON**	**LLys**	**HLys**	**SEM**	***P*-value**
Glycine	109.3	104.3	101.1	5.96	0.87
Alanine	607.0	630.8	668.1	68.38	0.94
Serine	68.42	77.55	76.85	7.392	0.87
Proline	54.79	66.78	57.48	3.861	0.44
Valine	400.6	467.7	468.4	39.81	0.76
Threonine	65.02	70.43	60.22	7.408	0.87
Hydroxyproline	33.41	35.44	36.38	2.926	0.93
Ornithine	101.0	141.5	148.2	18.56	0.57
Asparagine	31.69	35.57	34.09	2.979	0.88
Lysine	567.7	618.8	671.4	47.78	0.71
Glutamine	448.7	516.7	542.7	46.43	0.73
Glutamic acid	61.09	70.79	70.53	5.391	0.73
Methionine	24.71	31.5	29.73	3.046	0.67
Histidine	88.28	100.4	106.6	9.12	0.74
Hydroxylysine	1.174	1.513	1.281	0.1372	0.63
Phenylalanine	83.61	111.4	115.4	11.17	0.48
Arginine	92.91	125.8	119.2	13.54	0.61
Citrulline	72.05	107.6	101.8	9.34	0.27
Tyrosine	66.35	37.64	39.58	2.491	0.48
Tryptophan	66.35	82.49	84.70	9.282	0.71
Total amino acid (mmol/L)	3.299	3.632	3.708	3.3946	0.85

Values are the mean of five replicates (*n* = 5), with standard error of the mean (SEM).

CON = control group; LLys = with supplementary 5 g/d rumen-protected lysine; HLys = with supplementary 10 g/d rumen-protected lysine.

### 3.3 Rumen-protected lysine supplementation altered fecal VFAs concentration and microbial composition and community

The concentrations of acetate, propionate and the total volatile fatty acids in the feces of sika deer in the LLys group were significantly higher than those in the CON group (*P* < 0.05, Table 4).

**Table 4:** Comparison of volatile fatty acids in feces of sika deer in three groups.

**Item (mmol/L)**	**CON**	**LLys**	**HLys**	**SEM**	***P*-value**
Acetate	40.63^b^	71.73^a^	52.98^a^^b^	5.531	0.04
Propionate	9.50^b^	18.31^a^	13.23^a^^b^	1.689	0.06
Isobutyrate	0.75	0.97	0.99	0.553	0.10
Butyrate	6.19	5.85	4.69	0.478	0.43
Isovalerate	1.43	1.60	1.17	0.397	0.89
Valerate	2.20	2.04	1.27	0.347	0.46
Total VFAs	60.70^b^	100.07^a^	74.33^a^^b^	7.751	0.69

Values are the mean of five replicates (*n* = 5), with standard error of the mean (SEM).

CON = control group; LLys = with supplementary 5 g/d rumen-protected lysine; HLys = with supplementary 10 g/d rumen-protected lysine.

a, b means within a row with different superscript letters differ significantly from each other (*P* < 0.05).

**Figure 1:**
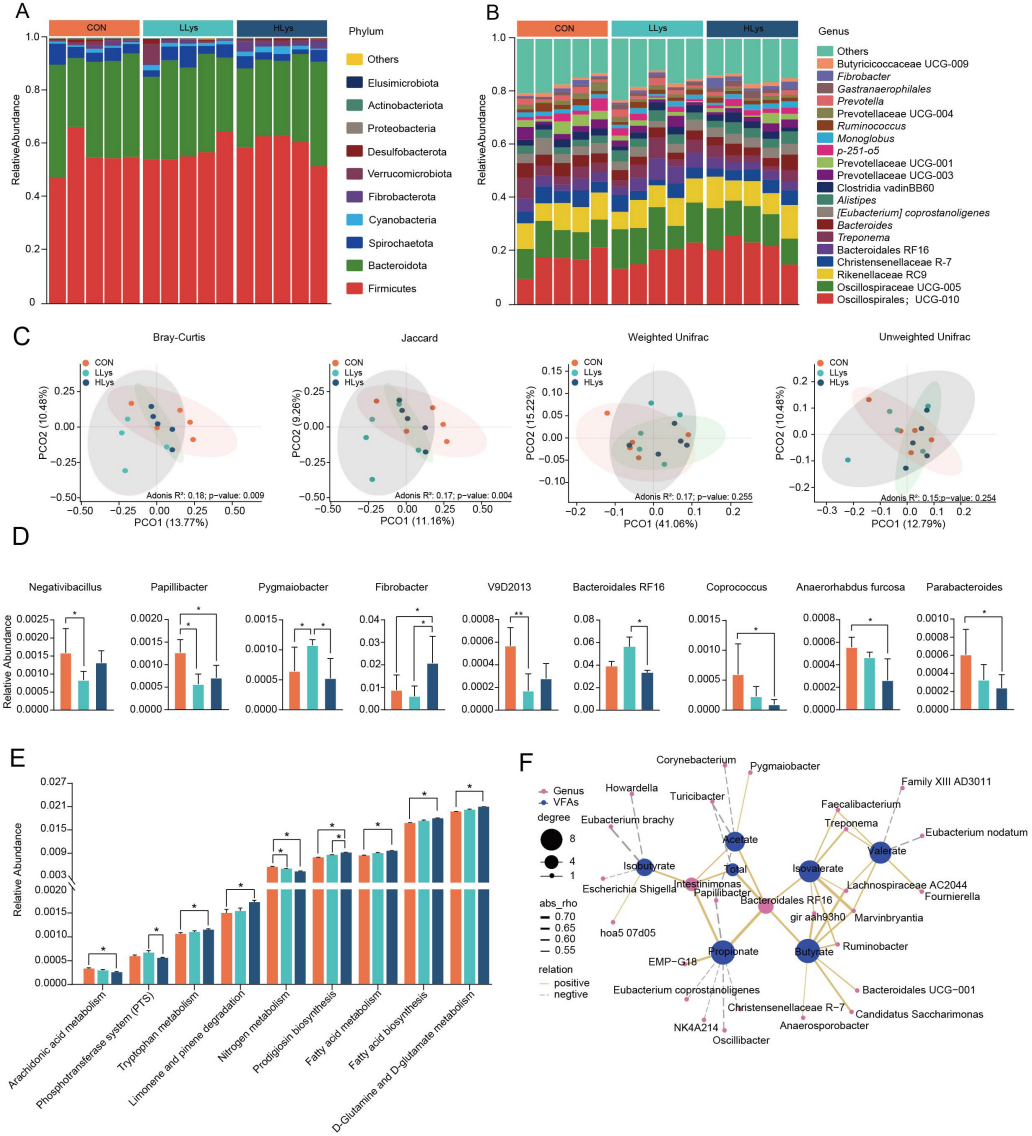
The community composition and functional analysis of fecal bacteria in three groups. The community composition of fecal bacteria in three groups at **(A)** phylum and **(B)** genus levels. **(C)** Beta diversity analysis, **(D)** Differential bacterial genera, **(E)** Differential bacterial functions at KEGG level 3. **(F)** Correlation analysis between bacterial relative abundance and volatile fatty acids. ^*^P < 0.05.

The results showed an average of 104,478 qualified sequences were obtained for each sample, and these sequences were denoised using the DADA2 method to obtain ASVs (Amplicon Sequence Variants). Following the exclusion of data related to archaea, unknown organisms, and those with no blast hit, a total of 3,639 ASVs were identified. A total of 24 phyla were identified in the fecal samples across the three groups. Firmicutes was the predominant bacterial phylum across all three groups, accounting for at least 55.73% of the total bacterial community in the feces, followed by Bacteroidetes and Spirochaetota, accounting for at least 31.83% and 3.24%, respectively (Figure 1A). At the genus level, the dominant bacterial genera in the feces of the three groups were *UCG-010* (order Oscillospirales) (CON = 16.61 ± 1.94%; LLys = 18.63 ± 1.88%, HLys = 21.31 ± 1.82%), *Oscillospiraceae UCG-005* (CON = 11.31 ± 0.64%; LLys = 13.68 ± 1.30%, HLys = 12.93 ± 1.00%), *Rikenellaceae RC9* (CON = 9.23 ± 0.68%; LLys = 8.99 ± 0.75%, HLys = 9.88 ± 1.02%). *Christensenellaceae R-7* (CON = 5.11 ± 0.51%; LLys = 4.27 ± 0.75%, HLys = 4.40 ± 0.45%) and *Bacteroidales RF16* (CON = 3.90 ± 0.43%; LLys = 5.64 ± 0.86%, HLys = 3.33 ± 0.21%) (Figure 1B).

We further revealed the differences in bacterial community across three groups. The results showed non-significant differences of α-diversity (Supplementary Figure S1B) in three groups, and the PCoA showed a significant separation in microbial community structures based on Bray-Curtis distance (Adonis: *P* = 0.009) and Jaccard distance (Adonis: *P* = 0.004, Figure 1C). Nine genera showed significant differences across three groups (Figure 1D). The relative abundance of *Fibrobacter* was significantly increased in the HLys compared to CON, and the relative abundances of *Papillibacter, Coprococcus, Anaerorhabdus furcosa*, and *Parabacteroides* were significantly decreased (*P* < 0.05) in HLys than those in CON. We next applied PICRUSt2 to predict the potential functions of fecal bacteria across three groups. Based on KEGG level 3, the relative abundances of tryptophan metabolism, limonene and pinene degradation, prodigiosin biosynthesis, fatty acid metabolism, fatty acid biosynthesis, and D-Glutamine and D-glutamate metabolism were significantly increased in the HLys group compared to the CON group. The relative abundances of arachidonic acid metabolism and nitrogen metabolism were significantly decreased (*P* < 0.05, Figure 1E) in HLys than those in CON.

We further applied correlation analysis to indicate the association between bacterial relative abundance and VFAs concentration, the concentration of acetate was significantly positively correlated with the relative abundance of *Pygmaiobacter* and negatively correlated with *Turicibacter* and *Corynebacterium*. Propionate concentration was positively correlated with *EMP-G18* and negatively correlated with *NK4A214, Oscillibacter, Christensenellaceae R-7*, and *Eubacterium coprostanoligenes*. Additionally, the relative abundance of *Intestinimonas* and *Bacteroidales RF16* show positive correlations with both acetate and propionate, whereas *Papillibacter* shows negative correlations with both acids (Figure 1F).

### 3.4 Rumen-protected lysine supplementation altered blood gene expression and pathway function

**Figure 2:**
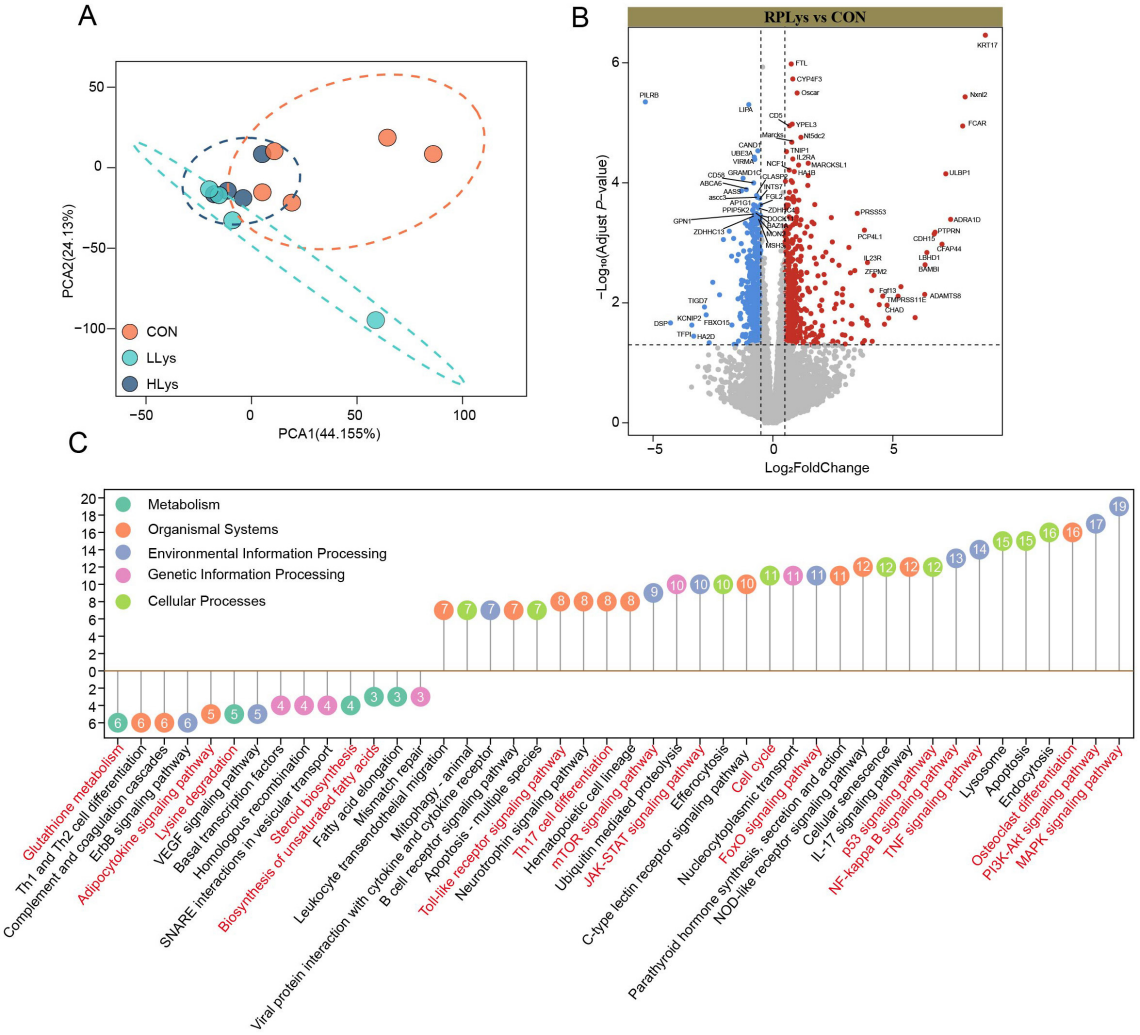
Rumen-protected lysine alters gene expression in the blood of sika deer (Con = 5, LLys = 4, HLys = 4). **(A)** PCA analysis illustrates the differences in gene expression across the three groups. Volcano plots display the up- and downregulated DEGs between the rumen-protected lysine-supplemented groups and the CON group **(B)**. Red and blue dots indicate up- and downregulated DEGs (*P* < 0.05 and |log_2_ (FC)| > 0.5). FC = fold change. The enriched KEGG pathways **(C)** of the up-and down-regulated DEGs in the rumen-protected lysine-supplemented groups compared to the CON group. Numbers in circles indicate the number of DEGs. Font in red highlight the pathways related to the effects of lysine on antler growth.

We performed transcriptomics to determine the gene transcriptional changes in the blood after rumen-protected lysine supplementation. The PCA results showed that the CON group was clearly separated from the other groups, whereas there was some overlap between the LLys group and the HLys group (Figure 2A). To demonstrate the effect of lysine supplementation on the expression of genes in the blood of sika deer, we focused on genes commonly upregulated or downregulated in both LLys and HLys groups compared to the CON group. We identified 1,232 DEGs (580 upregulated and 652 downregulated) in the blood of sika deer supplemented with rumen-protected lysine. Among them, upregulated DEGs included *KRT17, YPEL3, BAMBI, ADRA1D, FGF13, CP4L1, FCAR, TULBP1, CD5*, and *CYP4F3*, while downregulated DEGs included *PILRB, KLRI1, LIPA, GRAMD1C, AASS, AASDHPPT, DSP, KCNIP2*, and *TFPI* (Figure 2B).

According to the KEGG enrichment analysis, upregulated DEGs were enriched in 30 pathways including MAPK signaling pathway, PI3K-Akt signaling pathway, Apoptosis, TNF signaling pathway, p53 signaling pathway, FoxO signaling pathway, Cell cycle, JAK-STAT signaling pathway, NF-κB signaling pathway, Toll-like receptor signaling pathway, B cell receptor signaling pathway, Th17 cell differentiation, IL-17 signaling pathway, and Osteoclast differentiation. In contrast, the downregulated DEGs were enriched in 14 pathways including glutathione metabolism, lysine degradation, fatty acid elongation, and biosynthesis of unsaturated fatty acids, Th1 and Th2 cell differentiation, complement and coagulation cascades, and adipocytokine signaling pathway (Figure 2C).

## 4 Discussion

### 4.1 Antler growth performance and nutrient digestibility

This study showed that adding rumen-protected lysine significantly increased velvet antler production performance (final weight and the average daily gain) and nutrient apparent digestibility (CP, NDF and ADF) in sika deer. To date, few studies have investigated the effects of rumen-protected lysine addition on velvet antler growth in adult deer. Previous studies in fallow deer reported no significant improvement in velvet antler growth with rumen-protected lysine supplementation at a dose of 5 g/day (6), especially during the first antler cycle. In contrast, our results in adult sika deer showed that 10 g/day significantly increased antler weight and growth rate. This discrepancy may reflect species-specific differences in nutritional requirements, growth physiology, or metabolic responses to amino acid supplementation (13, 25). Furthermore, fallow deer in earlier studies were typically younger and still undergoing body development, whereas the adult sika deer in our study had completed skeletal maturation, potentially allowing for greater nutrient allocation toward antler growth. A similar result has also been observed in dairy cows, where milk production during the first lactation is influenced by ongoing body development (26). The body weight showed a positive correlation with the antler weight, which is consistent with observations in fallow deer (6). Moreover, our observation aligns with a previous study in goats, which reported that supplementation with rumen-protected lysine enhances the apparent digestibility of crude protein (27). This effect is likely due to lysine being one of the major limiting amino acids in corn-soybean meal-based diets (25, 28), as its addition helps balance the amino acid profile and improves CP utilization (29). Another important finding in this study is that rumen-protected lysine supplementation significantly increased the NDF and ADF apparent digestibility in sika deer. To date, no confirmed reports have demonstrated that NDF and ADF digestibility can be increased by rumen-protected lysine supplementation in ruminants, especially in the *Cervidae*. It is widely acknowledged that dietary protein increases can improve cellulose utilization or influence the fiber degradation bacteria in the gastrointestinal tract (30), and similar results have been found with rumen-protected arginine and methionine supplementation in sika deer (31). Another major finding of the present study was that rumen-protected lysine supplementation increased the apparent digestibility of calcium in sika deer, which further supports the fact that lysine promotes calcium absorption in the intestinal (32). These findings suggest that the effects of lysine supplementation on antler growth may be mediated through the increased serum protein and calcium levels, and enhanced fiber metabolism in the hindgut.

### 4.2 Serum biochemistry and amino acid metabolism

We first focused on the serum protein concentration of the sika deer. However, no significant changes were observed in serum protein levels in the host, the levels of BUN showed a concentration-dependent significant increase with the addition of lysine, suggesting that lysine supplementation increased protein catabolism in the sika deer (33). To clarify whether protein catabolism was influenced by rumen-protected lysine supplementation, we further examined free amino acids in the serum of sika deer. Serum-free amino acids also showed no statistically significant changes; however, aspartic acid, glutamic acid, proline, and arginine, which have been reported to be highly enriched in antlers (34), exhibited an increasing trend, especially in total free amino acids. These results suggested that lysine addition affected host amino acid metabolism and thus provided essential components for antler growth. Moreover, we then measured the concentration of serum calcium and observed a minimally concentration-dependent increase in the LLys and HLys groups compared to the CON group. However, a limitation of the present study is the small sample size, which may have reduced the statistical power to detect significant differences among the three groups. As the most prevalent element in antlers, increased calcium metabolism and circulating calcium levels in the host also provided an important substrate for antler growth (35).

### 4.3 Fecal volatile fatty acids and bacterial composition

Fiber degradation in the hindgut may be influenced by the rumen-protected lysine supplementation in sika deer. Changes in VFA concentrations, the key metabolites of microbial fiber fermentation (36), were important to reflect fiber digestion (37). Our results showed that the concentrations of acetate and propionate were significantly increased with rumen-protected lysine supplementation. Although the site of fermentation differs, rumen-protected lysine supplementation in combination with methionine increased the production of isobutyrate and isovalerate in the rumen (7). Notably, the LLys group exhibited higher acetate, propionate and total VFAs concentrations than the HLys group, suggesting a potential non-linear response to lysine dosage. One possible explanation is that moderate lysine supplementation may better support microbial fermentation in the hindgut (9), whereas higher doses could induce microbial shifts or adaptation that limit further VFA production. Although the exact mechanism remains unclear, this trend highlights the complexity of amino acid-microbiota interactions in the hindgut environment. To understand the variation in VFAs, we performed 16S rRNA sequencing to detect bacterial composition and communities in the feces of sika deer. Our results showed that Firmicute, Bacteroidota and Fibrobacterota were the predominant phyla and *UCG-010* (order Oscillospirales), *Oscillospiraceae UCG-005, Rikenellaceae RC9, Christensenellaceae R-7*, and *Bacteroidales RF16* were predominant genera across all three groups. Although Bray-Curtis and Jaccard distance analyses revealed significant differences in community composition among the three groups, while the α diversity index showed no significant differences between the LLys, HLys, and CON groups. This suggests that these compositional differences did not substantially affect the dominant microbial community in the hindgut of sika deer. Therefore, to reveal the changes in bacterial abundance among the three groups, we then continued to analyze the differences in the bacterial genus. These findings indicate a shift in the composition of fiber-degrading bacteria in the hindgut of sika deer, as evidenced by the increased abundances of Fibrobacter and *Bacteroidales RF16*. Our results showed a significant increase in the abundance of these fiber-degrading bacteria with the addition of rumen-protected lysine and supported the improvement in NDF and ADF digestibility (38). Additionally, we found that the relative abundances of *Anaerorhabdus furcosa* (39) and *Papillibacter* (40) were significantly higher in the CON group compared to the HLys group. Studies have shown that these bacteria are all associated with protein degradation. Functional predictions indicated that rumen-protected lysine supplementation affected tryptophan, D-glutamine-D-glutamate and nitrogen metabolism in hindgut bacteria, suggesting that increased protein digestion in sika deer may be highly correlated with changes in hindgut bacteria, but the contribution of these bacteria and their molecular mechanisms in protein digestion and metabolism of sika deer remains to be investigated based on metagenomic analysis. To further understand the relationship between the changes in bacterial abundance and variations in acetate and propionate concentrations, we performed a Pearson correlation analysis between them. The results indicate that different bacterial communities may play distinct roles in the production and metabolism of acetate and propionate. The relative abundances of *Intestinimonas* and *Bacteroidales RF16* both show positive correlations with acetate and propionate concentrations, suggesting that rumen-protected lysine supplementation through these bacteria further influences the production of VFAs. In contrast, the relative abundance of *Papillibacter* is negatively correlated with both acetate and propionate, possibly because *Papillibacter* is a butyrate producer of VFAs in the gut (41). It should be noted that VFA concentrations and bacterial composition in this study were based on fecal samples, which primarily reflect hindgut fermentation. While appropriate for assessing the effects of rumen-protected lysine, this approach does not capture rumen-level microbial activity and thus represents a limitation. Future studies including both rumen and hindgut samples are needed to provide a more complete picture of gastrointestinal microbial fermentation.

### 4.4 Blood gene expression and functional pathways

Subsequently, we performed transcriptomic analyses to examine whether the genes were differentially expressed in blood after rumen-protected lysine supplementation in sika deer. The results of PCA clearly showed that the LLys and HLys groups were separated from the CON group, indicating gene expression was changed by rumen-protected lysine supplementation in sika deer. Then, we identified 1,232 genes significantly altered between the CON and rumen-protected lysine-treated groups. Among them, we focused on those genes with higher upregulation multiples and found that *KRT17* (42), *YPEL3* (43), and *BAMBI* (44) were related to cell proliferation, and *ADRA1D* (45), *FGF13* and *PCP4L1* (46) were involved in calcium signaling, and genes associated with immune function including *FCAR* (45), *ULBP1* (47), *CD5* (48) and *CYP4F3* (49). The downregulation genes *PILRB, KLRI1* and *KLRB1* were also involved in immune function, and the first and last two genes, including *LIPA* (50), *GRAMD1C* (51), *AASS* (52) and *AASDHPPT* (53) are associated with lipid metabolism and amino acid metabolism, respectively. Importantly, antler is a rapidly regenerating organ characterized by intense mesenchymal proliferation, chondrogenesis, and endochondral ossification (1). The significantly upregulated genes *KRT17* and *YPEL3*, which are involved in cell cycle regulation, may indicate increased mesenchymal expansion at the antler tip, where active cartilage and bone formation occurs (42, 43). Moreover, the upregulation of *ADRA1D* and *FGF13*, both implicated in calcium signaling pathways, suggests a potential enhancement of intracellular calcium mobilization and modulation of calcium ion flux across cellular membranes (45, 46). These molecular events are essential for the activation of calcium-dependent enzymatic systems and matrix mineralization processes, which are critical for the progression of chondrogenesis to ossification during the advanced stages of antler development (35). These results together suggested that lysine supplementation may regulate cell proliferation, calcium absorption and transport to influence the antler growth in sika deer through these target genes.

Therefore, we conducted a KEGG enrichment analysis on the DEGs and focused on the above functions. The results showed that lysine may regulate cell proliferation through the MAPK signaling pathway, PI3K-Akt signaling pathway, Apoptosis, TNF signaling pathway, p53 signaling pathway, FoxO signaling pathway, Cell cycle and JAK- STAT signaling pathway regulates cell proliferation. Several reports have revealed that lysine nutrition improves the acetylation of lysine by providing higher amounts of lysine residues and influences histone modifications which activate the transcription of the p53 gene (54, 55), which plays a key role in cell cycle regulation, DNA damage repair, and apoptosis (56). Our results showed that the upregulated genes were significantly enriched in the p53 signaling pathway, and higher upregulation genes (*YPEL3* and *KRT17*), which were targeted by p53 (13, 43), also showed a significant increase, indicating the potential roles of target gene transcription and pathway activation in cell proliferation with lysine supplementation. Moreover, the results also showed that DEGs significantly enriched the NF-κB signaling pathway Toll-like receptor signaling pathway, B cell receptor signaling pathway, Th17 cell differentiation and IL-17 signaling pathway. Among them, IL-17 can activate the IKK complex by binding to IL-17R and initiating the recruitment of downstream adapter molecules (such as *ACT1*), leading to phosphorylation, release, and translocation of NF-κB to the nucleus, inducing the expression of inflammation-related genes (such as *IL6, TNF, CXCL8*, etc.) (57). Moreover, the NOD signaling pathway can activate immune and inflammatory responses through the NF-κB and MAPK pathways (58). The BCR signaling pathway activates the IKK complex, activating NF-κB and promoting the survival and differentiation of B cells into antibody-secreting cells (59). These findings together suggested that NF-κB plays a central role in multiple immune signaling pathways in the present study. A strong relationship between lysine and NF-κB has been reported that lysine can affect the activation and transcriptional regulation of NF-κB by acetylating the main subunit and increasing its binding to the promoter region of target genes in the nucleus (60). Moreover, *FCAR, CD5* and *CYP4F3* are also associated with the activation of the NF-κB pathway. *FCAR* encodes the Fcα receptor (CD89), a receptor for immunoglobulin A, the binding activates tyrosine kinase-dependent signaling pathways and then activates the NF-κB signaling pathway, which promotes pro-inflammatory cytokine release (e.g. IL-6, TNF-α) (61). *CD5* and *CYP4F3* are known to negatively regulate the immune response by modulating the activation of T and B cells or indirectly by metabolizing the inflammatory mediator LTB4, which regulates the activation of the NF-κB pathway (62, 63).

Another major finding in this study was the genes that were related to calcium signaling pathways and regulation were significantly upregulated with lysine supplementation. *ADRA1D* transcription regulates Gq proteins to promote the production of phosphatidylinositol (IP3), which further acts on the IP3 receptor on the endoplasmic reticulum to promote intracellular calcium ion release, and plays an important role in the regulation of intracellular calcium levels and calcium-dependent physiological processes (64, 65). Studies have shown that *FGF13* can indirectly affect intracellular calcium homeostasis by regulating calcium ion inward and outward flow through modulation of voltage-gated sodium channels (66). Previous studies have shown that lysine can improve calcium absorption efficiency by increasing intestinal cell membranes' permeability to calcium ions and facilitating the movement of calcium ions from the intestines into the blood circulation (5). Thus, the above results together suggest that lysine addition significantly affected the calcium signaling pathway and also reveal the molecular mechanisms involved in increased calcium digestion and metabolism levels in the present study. Moreover, we found that genes and pathways associated with bone development were also significantly enriched in upregulated genes (*OSCAR* and enrichment pathway Osteoclast differentiation), suggesting that lysine significantly impacts bone development in the present study. Lysine has been shown to be involved in collagen synthesis by participating in a reticular process that nourishes collagen bundles, thus influencing bone formation and remodeling (67).

Moreover, rumen-protected lysine supplementation significantly affects amino acid metabolism (glutathione metabolism, lysine degradation and mTOR signaling pathway) and lipid metabolism (adipocytokine signaling pathway, steroid biosynthesis, biosynthesis of unsaturated fatty acids and fatty acid elongation) signaling pathways. In our earlier study of antler growth with the dietary addition of 25-Hydroxyvitamin D, we found an increase in antler weight after down-regulation of fatty acid elongation and unsaturated fatty acid biosynthesis pathway gene expression in the antler growth of sika deer, a possible explanation is that 25-Hydroxyvitamin D increased the calcium absorption, which is consistent with the present study, suggesting that calcium content increased may contribute to antler growth through a lipid metabolism pathway (4). Together, these results reveal that lysine addition probably affects the metabolic, developmental, and immune functions of the organism by regulating the expression of the above key target genes. However, further cellular and molecular studies are needed to investigate the mechanisms underlying the effects of these target genes on the relevant signaling pathways. It is important to note that the transcriptomic data in this study were derived from peripheral blood rather than directly from antler tissue. Blood transcriptome analysis provides valuable insights into the systemic physiological and immune responses of the host and is commonly used in animal studies due to its accessibility and stability. However, gene expression in blood may not fully capture local transcriptional activity at the antler growth site, such as region-specific signaling, chondrogenesis, or ossification processes. This limitation should be considered when interpreting the functional relevance of differentially expressed genes.

These findings also provide valuable implications for future research and practical applications in deer farming. Given the observed improvements in antler growth, nutrient digestibility, and systemic gene expression, future studies should aim to determine the optimal dosage and duration of rumen-protected lysine supplementation for different physiological stages or production goals. Moreover, considering the potential genetic variability among deer populations, further investigations in genetically diverse or larger-scale commercial herds would help to validate the generalizability of these findings. In addition, functional validation of the key microbial and host gene expression changes, particularly those related to cell proliferation, calcium signaling, and immune regulation, will be crucial for elucidating the mechanisms underlying antler development. Ultimately, this research supports the potential use of dietary lysine regulation as a nutritional strategy to enhance velvet antler yield, which has important economic significance in modern sika deer production systems.

## 5 Conclusions

Our results suggest that rumen-protected lysine supplementation (i) improves the final antler weight and increased the protein and calcium digestibility in sika deer; (ii) alters the fecal bacterial community composition and VFA profiles, particularly regarding fiber-degrading bacteria; (iii) affects the gene expression of the cell proliferation, calcium signaling, osteoclast differentiation, amino acids metabolism and immune response in blood of sika deer. Taken together, these observations provide a better understanding of the effects of rumen-protected lysine supplementation on sika deer.

## Data Availability

The datasets presented in this study can be found in online repositories. The names of the repository/repositories and accession number(s) can be found below: https://www.ncbi.nlm.nih.gov/, SRR31824852-SRR31824866; https://www.ncbi.nlm.nih.gov/, SRR31762789-SRR31762804.

## Supplementary material

Supplementary Figure S1 **(A)** The correlation between final body weight and antler weight. **(B)** Comparison of the alpha diversity indices across the three groups.
