# Supplementary material for: Effects of rumen-protected lysine on antler growth performance, fecal bacterial community, and blood gene expression in sika deer
Source: Front Vet Sci. 2025 Jul 11;12:1583605. doi: 10.3389/fvets.2025.1583605 (PMC12292497; doi:10.3389/fvets.2025.1583605)
Supplement: Supplementary file 1 [file Table_1.docx]

**Table S1**. Ingredients and chemical compositions of the experimental diets used in this study

| Ingredient, (g/100 g) DM | | Nutrient levels, % | |
| --- | --- | --- | --- |
|  |  |  |  |
| Corn silage | 30.00 | Dry Matter | 86.01 |
| DDGS | 7.00 | Crude protein | 20.02 |
| Corn grain | 34.00 | Ether Extract | 4.13 |
| Soybean meal | 17.50 | Neutral detergent fiber | 58.94 |
| Wheat bran | 10.00 | Acid detergent fiber | 32.54 |
| Urea | 0.50 | ME, MJ/kg | 10.78 |
| Premix^1^ | 1.00 |  |  |
| Total | 100.00 |  |  |

1. Formulated to provide (per kg of DM): 150 g of salt, 200 g of NaHCO_3_, 75 g of Ca, 20 g of P, 600 mg of Mn, 680 mg of Fe, 960 mg of Zn, 300 mg of Cu, 140,000 IU of vitamin A, 55,000 of vitamin D_3_, 700 IU of vitamin E, and 600 mg of niacin.
