# Supplementary figures and images for: Effects of rumen-protected lysine on antler growth performance, fecal bacterial community, and blood gene expression in sika deer
Source: Front Vet Sci. 2025 Jul 11;12:1583605. doi: 10.3389/fvets.2025.1583605 (PMC12292497; doi:10.3389/fvets.2025.1583605)

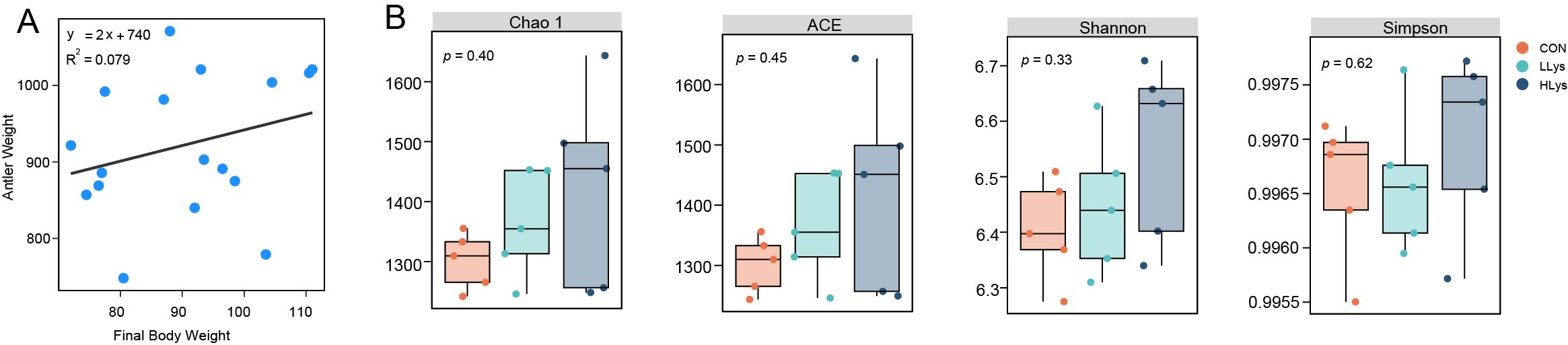

Supplement: Supplementary Figure S1 — (A) The correlation between final body weight and antler weight. (B) Comparison of the alpha diversity indices across the three groups. [file Image_1.jpg]
